# 220. Effectiveness of Piperacillin/tazobactam Loading Dose for Extended Infusion Dosing Among Patients with Gram-negative Bacteremia (EZ-LD)

**DOI:** 10.1093/ofid/ofad500.293

**Published:** 2023-11-27

**Authors:** Savan Patel, Sana Mohayya, Arsheena Yassin, Emily Aboujaoude, Judy Dao, Pinki Bhatt, Keith S Kaye, Ahmed Abdul Azim, Navaneeth Narayanan

**Affiliations:** Rutgers University, New Brunswick, New Jersey; Robert Wood Johnson University Hospital, New Brunswick, New Jersey; Robert Wood Johnson University Hospital, New Brunswick, New Jersey; Rutgers University, New Brunswick, New Jersey; Rutgers University, New Brunswick, New Jersey; Rutgers - Robert Wood Johnson Medical School, New Brunswick, New Jersey; Rutgers Robert Wood Johnson Medical School; Rutgers Robert Wood Johnson Medical School; Rutgers University Ernest Mario School of Pharmacy & Robert Wood Johnson University Hospital, New Brunswick, NJ

## Abstract

**Background:**

The rationale for a loading dose (LD) prior to administering extended infusion piperacillin/tazobactam (PTZ) is theoretical and based on pharmacokinetic/pharmacodynamic (PK/PD) principles and simulations. The clinical data to support the use of a LD for extended infusion are limited.

**Methods:**

This is a single-center, retrospective cohort study evaluating adult patients with gram-negative bacteremia who received extended infusion PTZ for at least 48 hours between 2015 and 2022. In December 2019, the study institution developed a policy that automatically ordered a PTZ LD whenever PTZ therapy was prescribed. The study compared patients who received the loading dose to those who did not. Electronic medical records were queried for patient demographic and clinical data. The primary endpoint was 30-day mortality. Key secondary endpoints included clinical cure, time to clinical cure, 14-day mortality, in-hospital mortality, time to mortality, and microbiologic cure. Clinical cure was defined by antibiotic discontinuation or narrowing of antibiotics in the setting of patient survival. Descriptive and inferential statistics were used for all data analyses. Logistic regression modeling was performed to control for confounding.

**Results:**

A total of 102 patients were included in the analysis, with 51 patients in the LD group and 51 patients in the no LD group. Baseline characteristics were similar in both treatment groups with the exception of ICU admission which was higher in the LD group (41% vs. 24%; P = 0.57). The 30-day mortality was 12% (6/51) in the LD group compared to 6% (3/51) in the no LD group (P = 0.49). There were no significant differences in 14-day mortality, in-hospital mortality, clinical cure, and microbiologic cure. After adjusting for potential confounders, there were no significant associations between LD and 30-day mortality (OR, 1.69; 95% CI, 0.36 to 7.96).

**Conclusion:**

We observed no significant association between LD for extended infusion PTZ and clinical outcomes. Larger cohorts with adequately powered sample sizes are needed to validate these findings. Future investigations should evaluate the impact of a LD for extended infusion PTZ on safety and PK/PD outcomes.

**Disclosures:**

**Pinki Bhatt, MD**, Sanofi: Grant/Research Support **Keith S. Kaye, MD, MPH**, Abbvie: Advisor/Consultant|Abbvie: Honoraria|Entasis: Advisor/Consultant|Entasis: Honoraria|GSK: Advisor/Consultant|GSK: Honoraria|Merck: Advisor/Consultant|Merck: Honoraria|Shionogi: Advisor/Consultant|Shionogi: Honoraria|VenatoRx: Advisor/Consultant|VenatoRx: Honoraria **Navaneeth Narayanan, PharmD, MPH, BCIDP**, Astellas: Honoraria|Beckman Coulter: Honoraria|Merck: Grant/Research Support|Shionogi: Grant/Research Support

